# Regulation of Insulin Receptor Trafficking by Bardet Biedl Syndrome Proteins

**DOI:** 10.1371/journal.pgen.1005311

**Published:** 2015-06-23

**Authors:** Rachel D. Starks, Andreas M. Beyer, Deng Fu Guo, Lauren Boland, Qihong Zhang, Val C. Sheffield, Kamal Rahmouni

**Affiliations:** 1 Department of Pharmacology, University of Iowa College of Medicine, Iowa City, Iowa, United States of America; 2 Department of Internal Medicine, University of Iowa College of Medicine, Iowa City, Iowa, United States of America; 3 Department of Pediatrics, University of Iowa College of Medicine, Iowa City, Iowa, United States of America; 4 Howard Hughes Medical Institute, University of Iowa College of Medicine, Iowa City, Iowa, United States of America; 5 FOE Diabetes Research Center, University of Iowa College of Medicine, Iowa City, Iowa, United States of America

## Abstract

Insulin and its receptor are critical for the regulation of metabolic functions, but the mechanisms underlying insulin receptor (IR) trafficking to the plasma membrane are not well understood. Here, we show that Bardet Biedl Syndrome (BBS) proteins are necessary for IR localization to the cell surface. We demonstrate that the IR interacts physically with BBS proteins, and reducing the expression of BBS proteins perturbs IR expression in the cell surface. We show the consequence of disrupting BBS proteins for whole body insulin action and glucose metabolism using mice lacking different *BBS* genes. These findings demonstrate the importance of BBS proteins in underlying IR cell surface expression. Our data identify defects in trafficking and localization of the IR as a novel mechanism accounting for the insulin resistance commonly associated with human BBS. This is supported by the reduced surface expression of the IR in fibroblasts derived from patients bearing the M390R mutation in the *BBS1* gene.

## Author Summary

A main function of the hormone insulin in the body is to regulate metabolism of glucose. The hormone causes body cells in different organs and tissues to utilize glucose from the bloodstream, storing the excess amount. Insulin resistance which reflects the inability of insulin to properly regulate glucose metabolism is common in people with obesity and/or type 2 diabetes. This insulin resistance is strongly associated with cardiovascular disease and increases the risk of death. However, the reasons that account for this insulin resistance phenomenon are currently not well understood. Here, we show that Bardet Biedl Syndrome proteins are required for proper action of insulin. We found that cells or animals that are deficient in Bardet Biedl Syndrome proteins are unable to respond to insulin. These results provide an explanation why patients that carry mutations in the Bardet Biedl Syndrome genes are insulin resistant, and will potentially contribute to understand common human forms of insulin resistance.

## Introduction

Insulin is critically involved in the regulation of glucose and lipid metabolism in various tissues ensuring the coordinated uptake and storage of the products of digestion. Insulin binding to its receptor initiates a myriad of intracellular events resulting in downstream activation of glucose uptake as well as glycogen, fatty acid, and DNA synthesis [1]. The insulin receptor (IR) is a dynamic molecule that moves through multiple cellular compartments throughout its lifecycle [2,3]. In its mature form, the IR resides in the plasma membrane as tetrameric proteins consisting of two extracellular, α-subunits, and two transmembrane, β-subunits. However, the molecular mechanisms underlying the trafficking of the IR to the plasma membrane remains largely unknown.

Bardet Biedl Syndrome (BBS) is a highly pleiotropic autosomal recessive disorder associated with clinical features that are considered the cardinal manifestations: obesity, retinal degeneration leading to blindness, postaxial polydactyly, learning disabilities and defects in the urogenital tract [4,5]. BBS is also associated with increased susceptibility to other disorders including insulin resistance and type 2 diabetes. Indeed, diabetes mellitus and the associated impairments in glucose metabolism and insulin sensitivity are common among BBS patients and often manifest during childhood [4,6–8,9]. Notably, even when matched for pubertal stage and body composition, individuals with BBS were found to exhibit significantly elevated insulin levels than controls [9,10].

Important mechanistic advances have been made in recent years in understanding the function of BBS proteins. Eight proteins [BBS1, BBS2, BBS4, BBS5, BBS7, BBS8, BBS9 and BBS18 (also known as BBIP10)] were found to form a stable complex, the BBSome [11]. Three other BBS proteins (BBS6, BBS10, BBS12) form another complex with CCT/TRiC family of group II chaperonins and mediate BBSome assembly [12,13]. Two additional BBS proteins, BBS3 (ARL6) and BBS17 (LZTFL1) interact with the BBSome and regulate its trafficking. In addition to the well-established role of BBS proteins in ciliary function, these proteins have been implicated in a number of cellular processes including intracellular trafficking, cell signaling and receptor trafficking [11,14–19].

In this study, we show that BBS proteins are necessary for the sorting of the IR and its cell surface expression. We found that the IR directly interacts with BBS17 and is present in a protein complex with the BBSome proteins. We further report that BBS proteins are required to maintain adequate levels of IR at the cell membrane and loss of BBS proteins leads to a reduction in the amount of IR at the cell surface. As a consequence, mice that lacks BBS proteins exhibit hyperglycemia, insulin resistance and blunted insulin-induced activation of IR signaling in insulin sensitive tissues (liver, skeletal muscle and adipose tissue). Notably, insulin resistance in BBS mice appears intrinsic and independent from obesity. These data identify BBS proteins as critical regulators of glucose metabolism and insulin sensitivity through the control of IR trafficking to the cell membrane. These findings also point to defects in IR trafficking as a mechanism of insulin resistance associated BBS.

## Results

### Direct interaction between the IR and BBS proteins

**Fig 1:**
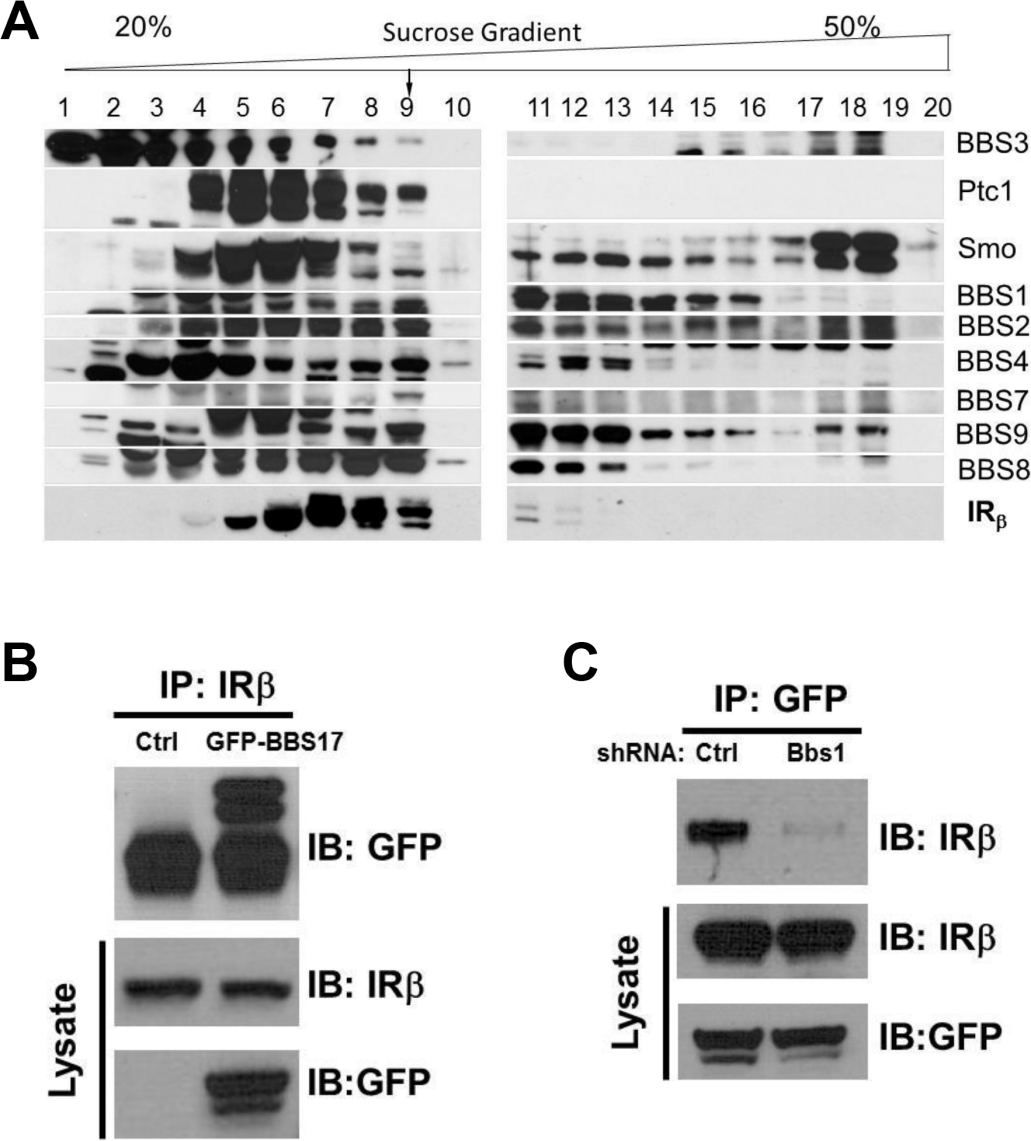
Physical interaction between BBS proteins and IR. **A**) Sedimentation analysis showing the β subunit of the IR in complex with the BBSome (corresponding to fraction 9). **B-C**) Reciprocal co-immunoprecipitation with GFP-tagged BBS17 and the β subunit of the IR in protein lysates from HEK293T cells. The interaction between the IRβ subunit and BBS17 can be detected whether IR antibody is used for immunoprecipitation (A) or GFP-BBS17 is used to precipitate IR (Ctrl line in C). Silencing the *Bbs1* gene with shRNA disrupt the interaction between BBS17 and IRβ (right line in C).

To investigate the possibility that BBS proteins are involved in IR trafficking, we first examined whether BBS proteins interact with the IR in a sucrose gradient fractionation experiment. Interestingly, a portion of the β subunit of the IR was identified in the same pool of proteins that contained the BBSome complex (Fig 1A). This fraction also contained other known partners of the BBSome such as Ptc1 and Smo proteins [20]. Next, we performed a pairwise reciprocal immunoprecipitation assay to investigate whether BBS proteins interact directly with the IR. Among all the BBS proteins tested, a physical interaction between the IR and BBS17 was identified based on the ability of the endogenous IR and GFP- or Flag-BBS17 to pull down each other in cells (Figs 1B and S1A). Similar results were obtained with the endogenous proteins (IR and BBS17) using mouse tissue (S1B Fig). Of note, shRNA-mediated reduction in the expression of BBS1 (S1C Fig), a key component of the BBSome, disrupted the interaction between BBS17 and the IR (Fig 1C). Together, these data indicate a direct interaction between BBS proteins and IR.

### BBS proteins are necessary for surface expression of the IR

**Fig 2:**
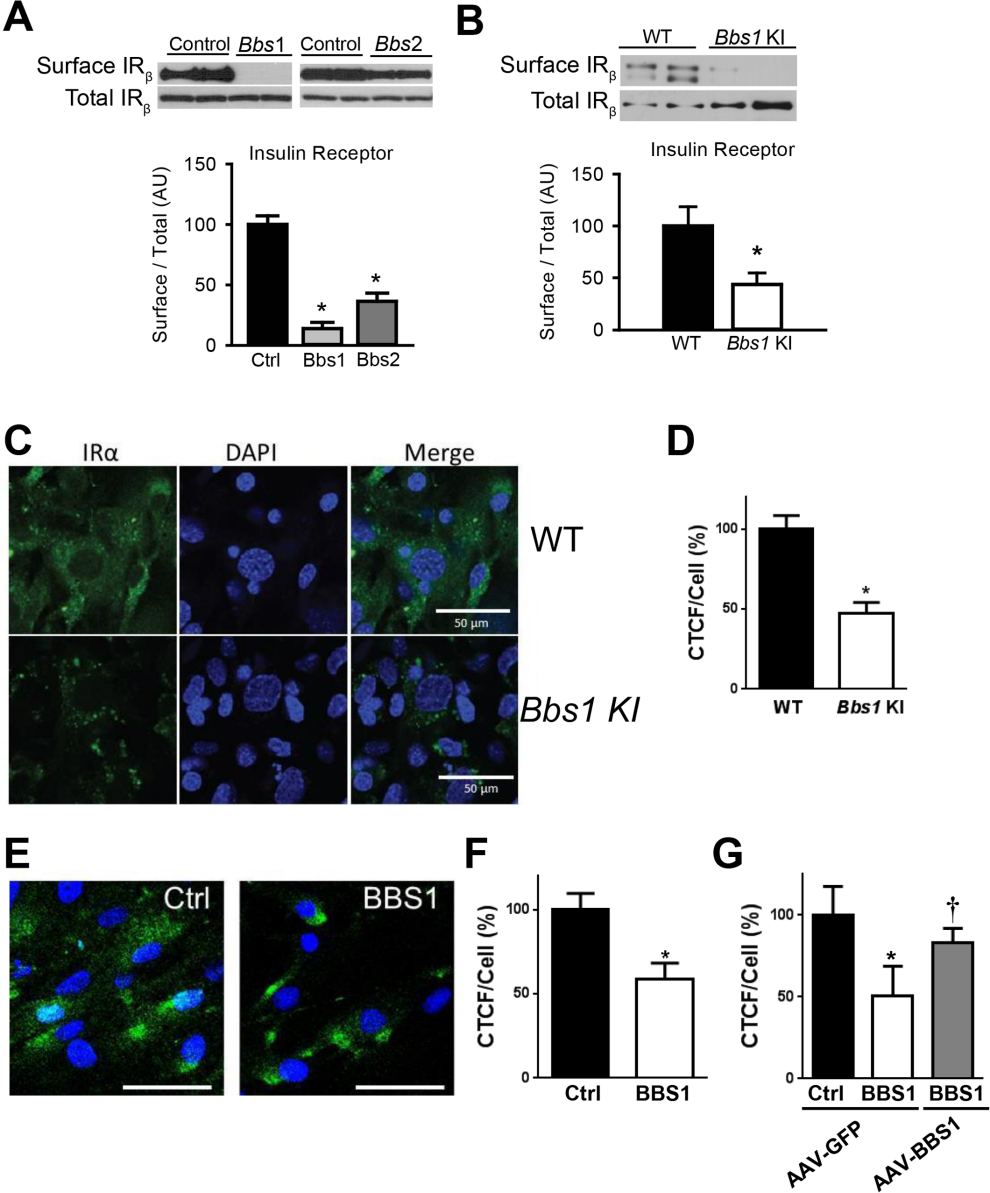
BBSome proteins are required for surface expression of the IR. **A**) Silencing the *Bbs1* or *Bbs2* genes using shRNA reduced IR surface expression in HEK293T cells. **B**) MEF cells obtained from *Bbs1* ^*M390R/M390R*^ knock-in (KI) mice have decreased membrane IR relative to wild type (WT) littermates as assessed by Western blot. **C-F**) Non-permeabilized *Bbs1* ^*M390R/M390R*^ MEF cells (**C-D**) and human *BBS1* ^*M390R/M390R*^ fibroblasts (**E-F**) have reduced surface IR (green) by immunostaining. **G**) AAV-mediated expression of wild type BBS1 protein rescues the defect in IR trafficking in human *BBS1* ^*M390R/M390R*^ fibroblasts. *P<0.05 vs. WT or Ctrl; †<0.05 vs. BBS1 cells treated with AAV-GFP all data are expressed as means ± SEM.

To test directly the requirement of BBS proteins for IR localization to the plasma membrane, we examined the effect of knocking-down expression of BBSome subunits on IR surface levels. In cells, shRNA-mediated silencing of either *Bbs1* or *Bbs2* genes (S1C and S1D Fig) significantly reduced the amount of IR present in the plasma membrane (Fig 2A). In contrast, there was no change in total IR protein expression. Next, we used mouse embryonic fibroblast (MEF) cells from mice bearing the M390R mutation in the *Bbs1* gene. This missense mutation, changing a methionine to an argine at position 390 of the BBS1 protein, accounts for about 80% of *BBS1* cases while *BBS1* mutations account for about 25% of all BBS cases [21]. We previously reported that *Bbs1* ^*M390R/M390R*^ mouse model phenocopy BBS [22]. Relative to littermate controls, *Bbs1* ^*M390R/M390R*^ MEF cells exhibited a significant decrease in the membrane fraction of the IR without significant change in total IR protein (Fig 2B). To assess whether this finding results from a specific defect in IR transport, we evaluated the levels of an unrelated membrane receptor, transferrin receptor. There was no significant difference in protein levels of transferrin receptor in the membrane fraction or total protein after *Bbs1* or *Bbs2* knock-down or in the *Bbs1* ^*M390R/M390R*^ MEF cells compared to control cells (S2 Fig).

The reduced amount of IR on the cell surface of *Bbs1* ^*M390R/M390R*^ MEF cells was further confirmed by immunohistochemistry. Using an antibody that recognizes an extracellular epitope of the α subunit of IR, we stained non-permeabilized MEF cells for IR on the surface of the cell. Notably, the intensity of the surface IR signal was significantly less in *Bbs1* ^*M390R/M390R*^ MEF cells as compared to control MEF cells (Fig 2C and 2D). There was also a difference in the pattern of staining. Wild type MEF cells exhibit typical plasma membrane staining, while *Bbs1* ^*M390R/M390R*^ MEF cells have a more punctate pattern indicating clustering of the few IRs found on the surface. Similarly, the surface level of the IR was reduced in cells in which expression of BBS chaperonin complex proteins (BBS6 and BBS12) were independently silenced using GFP-tagged shRNA (S3 Fig). Importantly, fibroblast cells obtained from *BBS1* ^*M390R/M390R*^ patients also exhibited reduced surface expression of the IR (Fig 2E and 2F). To test whether the altered trafficking of the IR can be rescued in *BBS1* ^*M390R/M390R*^ cells with a functional BBS1 protein we used an adeno-associated virus (AAV) vector expressing the wild type BBS1 protein reported previously [23]. The surface expression of the IR was recovered in the *BBS1* ^*M390R/M390R*^ cells treated with the AAV-*Bbs1* (Fig 2G) demonstrating that the altered trafficking of the IR in these cells is due to loss of function of the BBS1 protein.

### IR is not targeted to cilia

BBS proteins and complexes have been implicated in the trafficking of multiple cellular receptors, such as somatostatin receptor 3, dopamine receptor 1, vasopressin receptor 2, and neuropeptide Y family receptors, to cilia [16,17,19,24]. Thus, we tested the possibility that the IR may be trafficked to cilia. However, there was no overlap of the fluorescent labeling of the IR and cilia indicating that the IR is not targeted to the cilia (S4 Fig). Nonetheless, we cannot fully exclude the possibility that the IR in the cilia is below the level of detection or is targeted to the cilia of other cell types. Our finding is consistent with the concept that the IR relies on the BBSome for targeting to the plasma membrane, rather than to cilia.

### BBS mice are insulin resistant

**Fig 3:**
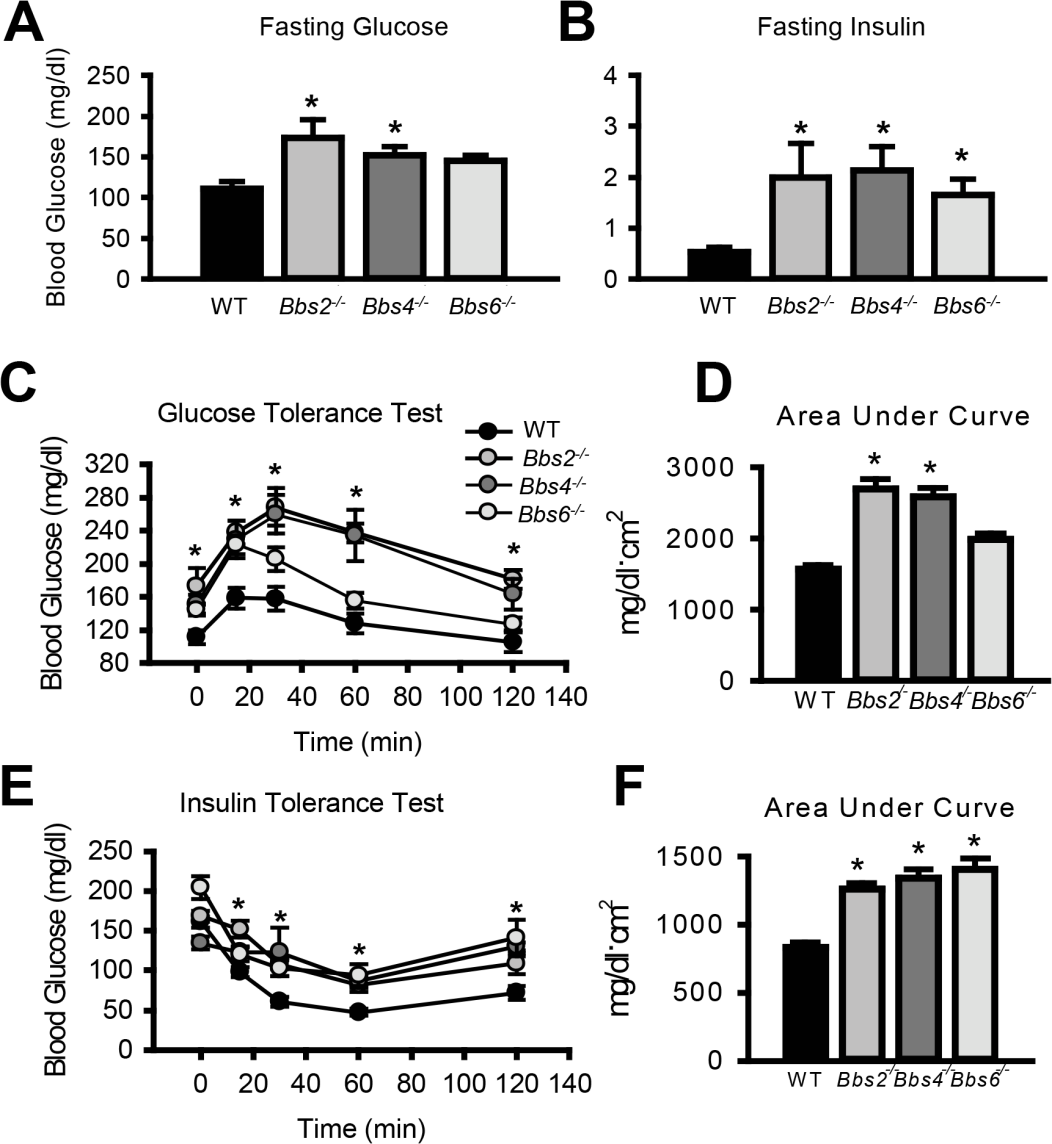
Insulin resistance and altered glucose metabolism in mice lacking functional BBS proteins. **A-B**) Obese Bbs2, Bbs4 and Bbs6 null mice are hyperglycemic (**A**), and hyperinsulinemic (**B**). Bbs2, Bbs4 and Bbs6 null mice exhibit glucose intolerance (**C-D**) and insulin resistance (**E-F**) relative to wild-type littermate controls. *P<0.05 vs. WT; data expressed as means ± SEM.

**Fig 4:**
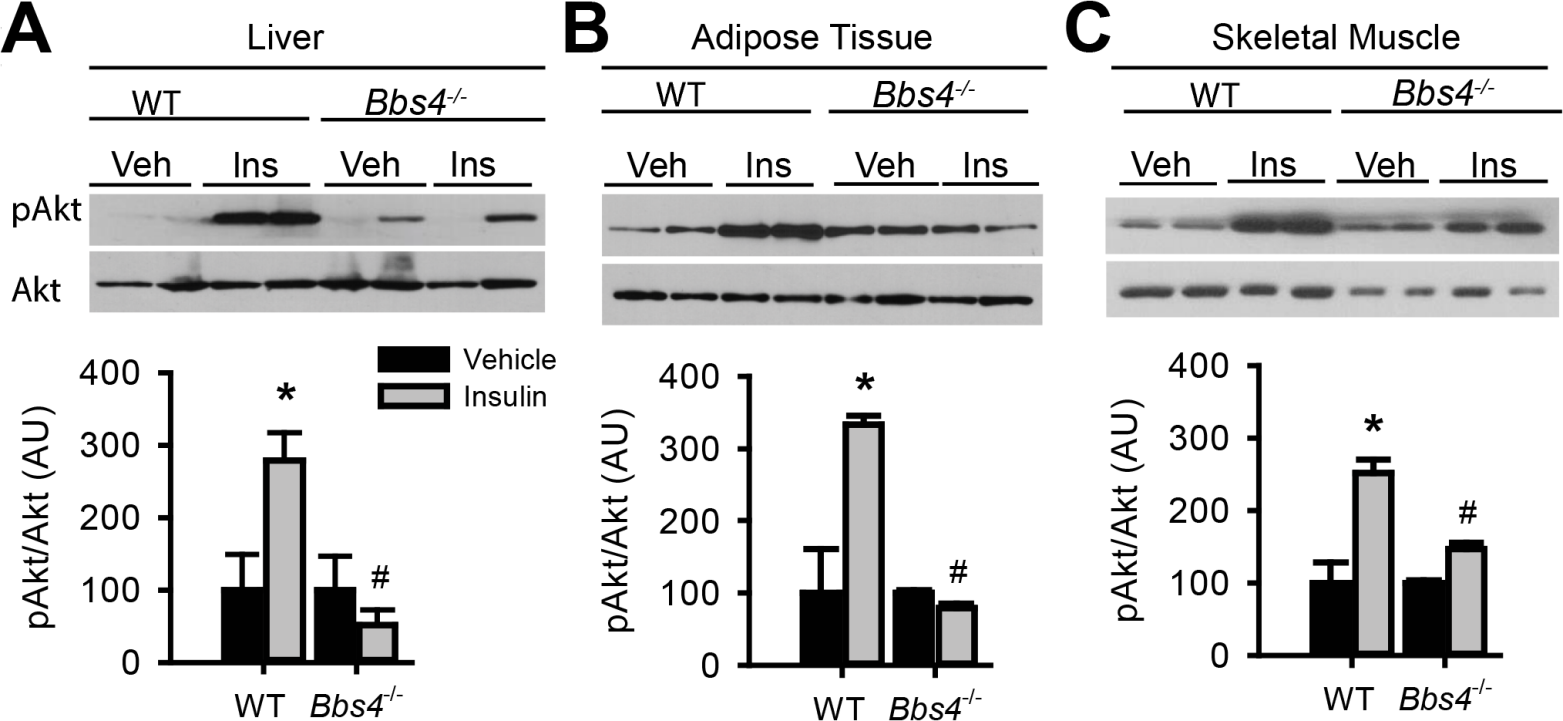
Obese Bbs4 null mice have reduced IR signaling (using phosphorylated Akt at S473 [pAkt] as readout), in liver (A), white adipose tissue (B) and skeletal muscle (C). *P<0.05 vs. Vehicle, # P<0.05 vs. WT-Insulin; data are expressed as means ± SEM.

Given the striking decrease in IR membrane levels caused by disruption of BBS proteins, we examined insulin sensitivity and glucose homeostasis in BBS mice. Consistent with a previous report [25], we found that *Bbs4* ^-/-^ mice are hyperglycemic (Fig 3A) and hyperinsulinemic (Fig 3B). *Bbs2* ^*-/-*^ and *Bbs6* ^*-/-*^ mice also displayed significantly elevated blood glucose and plasma insulin as compared to wild type littermates (Fig 3A and 3B). Further analysis using glucose and insulin tolerance tests showed that *Bbs2* ^*-/-*^, *Bbs4* ^*-/-*^, and *Bbs6* ^*-/-*^ mice are glucose intolerant (Fig 3C and 3D) and insulin resistant (Fig 3E and 3F). Next, we assessed whether the defects in glucose disposal and insulin action in BBS mice are associated with altered IR signaling (using Akt activation as a read out) in liver, skeletal muscle, and adipose tissue, the classic targets of insulin action [1]. In wild type mice, insulin treatment significantly increased the levels of phosphorylated Akt (pAkt) in all three tissues (Fig 4A–4C). In contrast, insulin did not significantly increase pAkt levels in liver, skeletal muscle and adipose tissue from *Bbs4* ^*-/-*^ mice. Importantly, our data demonstrate that BBS mice recapitulate the defects in glucose metabolism and insulin sensitivity reported in BBS patients.

### Insulin resistance in BBS mice is independent of obesity

**Fig 5:**
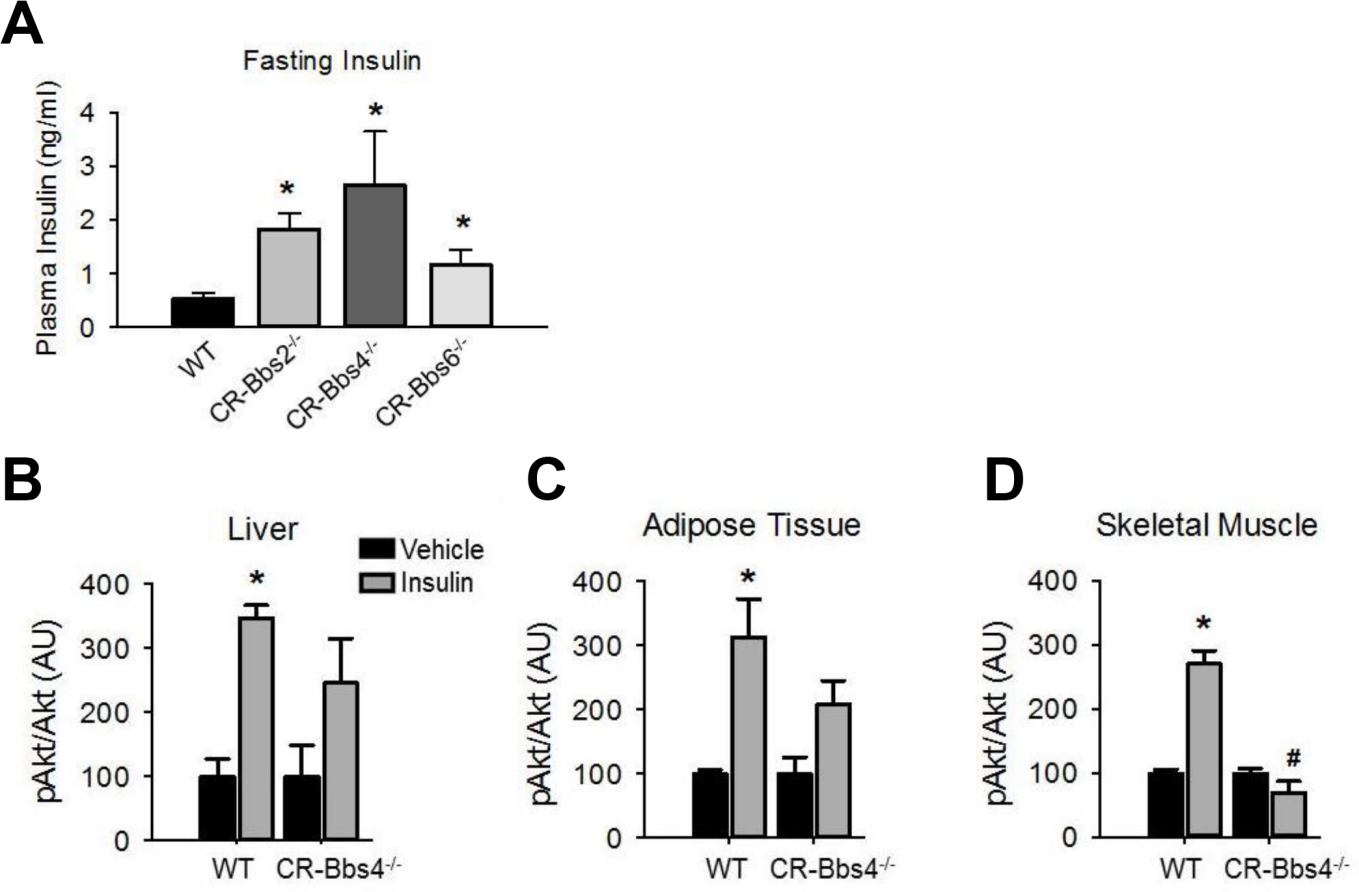
Alterations in insulin action and signaling in BBS mice is not related to obesity. **A**) Bbs2, Bbs4 and Bbs6 null mice rendered lean by calorie restriction (CR) are hyperinsulinemic. **B-D**) Lean Bbs4 mice have reduced IR signaling (pAKT^*S473*^) in liver (**B**), adipose tissue (**C**) and skeletal muscle (**D**). *P<0.05 vs. Vehicle, # P<0.05 vs. WT-Insulin; data are expressed as means ± SEM.

**Fig 6:**
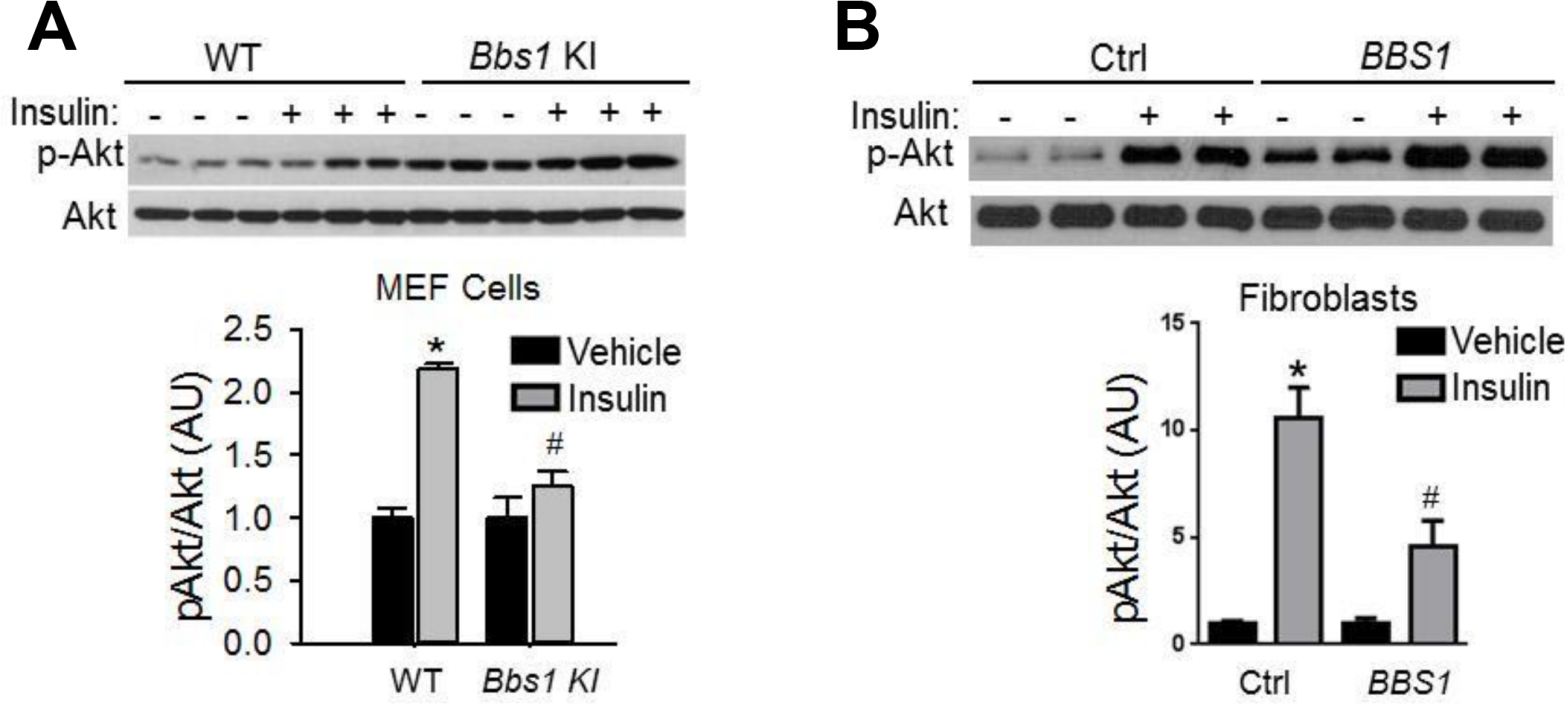
Blunted IR signaling in cells bearing mutant BBS1 protein. **A**) MEF cells obtained from *Bbs1* ^*M390R/M390R*^ mice have reduced Akt activation (pAKT^*S473*^) in response to insulin when compared to cells obtained from wild-type littermate controls. (**B**) Insulin-induced Akt activation was also blunted in fibroblasts derived from *BBS1* ^*M390R/M390R*^ patients relative to control cells. *P<0.05 versus WT or vehicle, ^*#*^P<0.05 versus WT-Insulin; all data are expressed as means ± SEM.

To examine whether the metabolic defects in BBS mice are secondary to obesity, we studied lean BBS mice that had their body weights normalized by calorie restriction (S5 Fig). Strikingly, the fasting insulin levels of calorie restricted *Bbs2* ^*-/-*^, *Bbs4* ^*-/-*^, and *Bbs6* ^*-/-*^ mice are significantly elevated (Fig 5A) indicating that the hyperinsulinemia associated with BBS is independent of obesity. Furthermore, insulin-induced Akt activation in the skeletal muscle, liver and adipose tissue was blunted in the calorie restricted *Bbs4* ^-/-^ mice relative to controls (Fig 5B–5D). Thus, the defect in insulin receptor signaling in *Bbs4* ^*-/-*^ mice is not related to obesity, but rather due to loss of BBS4 protein. These findings are consistent with the demonstration that surgery-mediated weight loss failed to suppress the hyperinsulinemia or manage glycemia in BBS patients [26,27]. It is also interesting to note that even when matched for pubertal stage and body composition, individuals with BBS exhibit elevated plasma insulin levels relative to the control subjects, which is consistent with the notion that insulin resistance associated with BBS is a primary defect [9]. This notion is further supported by the inability of insulin to activate Akt in *Bbs1* ^*M390R/M390R*^ MEF cells (Fig 6A). The *Bbs1* ^*M390R/M390R*^ MEF cells indicate the innate cellular response to insulin because they have never been exposed to either obesity or calorie-restriction. In addition, *Bbs1* ^*M390R/M390R*^ MEF cells provide further evidence from a model BBS that mimics human disease. Of note, *BBS1* ^*M390R/M390R*^ fibroblasts also exhibited blunted Akt activation in response to insulin (Fig 6B). These findings are in line with the reduced membrane fraction of the IR described above in these same cells.

## Discussion

The current study establishes BBSome proteins (BBS1, BBS2 and BBS4) and by implication the BBSome as key mediators of IR trafficking to the cell membrane. Additionally, BBS6 and BBS12 which are not part of the BBSome but are needed for BBSome formation are implicated as well which is consistent with the role of these proteins in the BBSome assembly. Interestingly, the role of BBS proteins in IR trafficking appears not to involve localization to cilia, indicating a more generalized role in intracellular trafficking as has been shown for retrograde trafficking of the melanosome transport in *bbs* knockdown zebrafish models [18]. Our study also indicates that defects in IR trafficking and localization are major mechanisms of insulin resistance in BBS.

Strikingly, very little is known about the molecular mechanisms underlying anterograde IR trafficking. It has been known that newly synthetized IR moves through multiple subcellular compartments of the cell before its insertion in the plasma membrane [2,3]. During this process the receptor undergoes various modifications including glycosylation which are important for its trafficking [28,29]. Here, we demonstrated that disruption of *Bbs* genes interfere with IR localization to the cell membrane. The precise steps and events by which the BBS proteins influence IR trafficking are not clear and will necessitate further studies. For example, a limitation of the current study is that it is unknown whether our finding of decreased IR in the plasma membrane is due to decreased trafficking of newly synthesized IR to the membrane as opposed to increased rate of loss of plasma membrane bound IR. Related to this and given that disruption of *Bbs* genes did not alter the overall expression levels of the IR it will be interesting to determine the cellular compartment where the receptor resides when the BBSome is impaired.

Proteins other than BBS have been shown to influence the transport of the IR to the surface. For example, a previous study [30] demonstrated the importance of myotonic dystrophy protein kinase for IR targeting to the plasma membrane in skeletal muscle cells which may explain the high prevalence of insulin resistance in patients with myotonic dystrophy [31]. The relationship between BBS proteins and myotonic dystrophy protein kinase in IR trafficking remain to be determined.

We considered the possibility that BBSome impairment interfere with overall transport of membrane receptors in a manner similar to what occur in cells infected with prions proteins [32]. However, the ability of the transferrin receptor to maintain its presence at the cell surface in the absence of BBS proteins indicates that overall transport of membrane receptors is not disrupted and that BBS proteins mediate the routing to the plasma membrane of specific receptors such as the IR.

To clarify the relevance of BBS proteins to whole body glucose metabolism and insulin action, we studied several mouse models that lack various BBS genes. Similar to the phenotype reported in patients, BBS mouse models displayed type 2 diabetes phenotype as evidenced by the hyperglycemia and hyperinsulinemia. Furthermore, these mouse models exhibited glucose intolerance and insulin resistance. The defective IR signaling as indicated by the blunted insulin-induced Akt activation further corroborates the insulin resistance phenotype. Cells obtained from BBS patients also exhibited blunted Akt stimulation in response to insulin and have reduced IR surface expression. Together, these findings establish the significance of BBS proteins for insulin sensitivity and glucose homeostasis. These data also indicate that disruption of IR trafficking underlie insulin resistance in BBS. The recent demonstration by Lim et al. [33] that polymorphism in *BBS* genes such as *BBS10* increase the risk of type 2 diabetes in a recessive state raise the possibility that *BBS* genes may contribute to the pathogenesis of common forms of type 2 diabetes perhaps through their role in IR handling.

It should be noted that in contrast to our findings, a previous report by Marion *et al*. showed a paradoxical improvement in insulin sensitivity in *Bbs12* ^-/-^ mice [34]. Indeed, *Bbs12* null mice were found to have enhanced adipogenesis, glucose tolerance and insulin sensitivity of adipose tissue whereas here we show that BBS12 is required for IR trafficking to the cell membrane. There is no clear explanation for the contrast between our findings and this previous report, but there are many factors that may have contributed including the heterogeneity of BBS and associated phenotypes, age and genetic background of the mice. Alternatively, BBS12 may have a function in adipocyte differentiation besides its typical role as part of the BBS chaperonin complex and mediating BBSome formation. In addition to their involvement in the BBS complexes individual BBS proteins could be engaged in other cellular tasks requiring further investigation. This possibility is supported by several evidences including the synergistic effects of suppressing *bbs* genes in zebrafish [35], and the variability of the phenotypes of BBS mice [36] and patients carrying mutations in BBS proteins that belong either to the same complex (e.g. chaperonin complex) or related complexes (e.g. chaperonin complex vs BBSome) [37].

In conclusion, the current study indicates that BBS proteins regulate glucose metabolism and insulin sensitivity through its involvement in the trafficking of the IR to the cell membrane. These findings also indicate that insulin resistance associated BBS arise from defective localization of the IR.

## Materials and Methods

### Animals

*Bbs1* ^*M390R/M390R*^, *Bbs2* ^-/-^, *Bbs4* ^-/-^, and *Bbs6* ^-/-^ mice were bred on mixed 129SvEv and C57B/6J backgrounds. As there was no evidence of sexual dimorphism, both male and female mice were used for each experiment. Wild type littermates were used as controls. Mice were housed at the University of Iowa Animal Care facility in a temperature and humidity controlled room on a 12 hour light/dark cycle with free access to food and water except in the calorie restriction experiment where food intake of BBS mice was restricted. At 6–8 weeks of age, which is before the onset of obesity, individually housed BBS mice were given 75–80% of the daily food intake of their littermate’s intake for 8 weeks as we reported previously [38,39]. All animal testing was performed based on guidelines set forth by the National Institutes of Health and approved by The University of Iowa Institutional Animal Care and Use Committee (Protocols 1211242 and 1301003). Euthanasia was performed with an overdose of anesthesia (Ketamine/xylazine cocktail) followed by the combination of thoracotomy and harvesting of vital organs (heart, liver and/or brain).

### Mouse embryonic fibroblast (MEF)

MEF were established from wild type and *Bbs1* ^*M390R/M390R*^ E13.5 embryos. Viscera, liver and heart were discarded from the embryos, and the remaining embryo was cut into fine pieces in the presence of Trypsin-EDTA (Invitrogen). Further Trypsin-EDTA was added and the digested tissue was incubated in a 15 ml tube at 37°C, 95% humidity and 5% CO_2_ for 15 min. The digested tissue was mixed with Dulbecco’s Modified Eagle’s Medium (DMEM, high glucose without sodium pyruvate), 10% heat-inactivated fetal bovine serum (FBS), non-essential amino acids, and 50 U/ml penicillin/streptomycin (Gibco) and passed through a 10 ml pipette 5 times. MEFs were cultured in a 100 mm culture dish for experiments.

### Human fibroblasts

All patients provided written, informed consent for this study, which was approved by the Institutional Review Board of the University of Iowa. Skin biopsies were collected and used for the generation of fibroblasts which were grown in DMEM with 10% FBS, and 1% sodium pyruvate at 37°C with 5% CO_2_ as described previously [40,41].

### Cell transfection

HEK293T cells were cultured in regular growth medium; DMEM supplemented with 5% (v/v) fetal bovine serum, and 1% (v/v) sodium pyruvate, at 37°C with 5% CO_2_. Two μg of plasmid DNA encoding a Flag-tagged BBS17 protein [42] or shRNA against *Bbs1*, *Bbs2*, or control [38] on an LKO1 plasmid or GFP expressing *Bbs6* and *Bbs12*-shRNA on an GIPZ plasmid was transfected into 70–80% confluent HEK293T cells in 60 mm dish with FuGENE 9 (Roche) according to manufacturer’s instructions. After 48 h incubation with the DNA/FuGENE mixture, cells were used for the surface proteins assays as described below or co-immunoprecipitation using endogenous IR or Flag-BBS17 for pull down or immunohistochemistry for surface IR alpha. Fibroblasts derived from control and BBS1 patients were infected with 1x10^5^ PFU of AAV expressing green fluorescent protein (GFP, used as control) or *Bbs1* for 48 h before processed for surface IR_α_ immunostaining.

### Biotin labeling assay

Biotin labeling was performed as previously described [43]. Cells were placed on ice and washed with phosphate buffered saline (PBS). EZ-link Sulfo-NHS-SS-Biotin (Thermo Scientific) was used to prepare biotinylation solution immediately before incubating cells for 30 min at 4°C. Cells were washed three times on ice and immediately lysed in lysis buffer (50 mM HEPES pH7.5, 137 mM NaCl, 1% NP-40, 0.25% Na-deoxycholate, 2 mM EDTA, 2 mM Na_3_VO_4_, 10 mM NaF, 10% glycerol, protease inhibitor cocktail [Roche complete Mini, EDTA-Free]) for 30 min at 4°C. The cell lysate was centrifuged at 14,000 *g* for 30 min at 4°C. Whole cell lysates were used for detection by immunoblot and for immunoprecipitation with Pierce Streptavidin UltraLink Resin (Thermo Scientific) overnight at 4°C to form Avidin-Biotin complex (ABC). The ABC resin was washed in PBS three times, and precipitated proteins were analyzed by immunoblotting.

### Sucrose gradient fractionation

Sucrose gradient assay was performed as previously described [13]. Briefly, protein lysates were centrifuged at 20,000 × *g* for 20 min. The supernatants were loaded onto a 20–60% sucrose gradient. The gradient was centrifuged at 100,000 × *g* for 14 h using a TH-660 rotor Thermo Scientific (Asheville, NC). Two hundred-microliter fractions were taken from the top and precipitated by cold acetone. Precipitated samples were spun at 20,000 × *g* for 15 min. The pellets were dissolved in SDS-PAGE sample buffer and used for Western blotting.

### IR signaling assay

Mice were fasted overnight in a clean cage. On the morning of the test, mice were weighed and anesthetized with xylazine-ketamine injections. Once the mice were anesthetized, the jugular vein was cannulated for either vehicle (physiological saline) or insulin (5 U/kg) injection. Mice were sacrificed after 15 min and liver, white adipose tissue pad and soleus muscle collected. Tissues were homogenized in lysis buffer (50 mM HEPES pH7.5, 137 mM NaCl, 1% NP-40, 0.25% Na-deoxycholate, 2 mM EDTA, 2 mM Na_3_VO_4_, 10 mM NaF, 10% glycerol, protease inhibitor cocktail [Roche complete Mini, EDTA-Free]). The extracts were centrifuged at 14,000 *g* for 30 min at 4°C. Protein concentration of the obtained supernatant was measured using the Bradford protein assay [44]. The level of phospho-Akt and total Akt was determined by Western blotting.

### Immunoblotting

Proteins in whole tissue lysate were resolved by 9% acrylamide SDS-PAGE, and the proteins were then transferred to PVDF membranes. Membranes were blocked in 5% nonfat dry milk in Tris-buffered saline (NaCl, KCl, Tris-base) with 0.1% Tween-20 (TBST) for 1 h at 25°C then incubated with primary antibodies at 4°C overnight. Membranes were further incubated with secondary antibodies conjugated with horseradish peroxidase (HRP) for 2 h at 25°C. Visualization was performed with enhanced chemiluminescence (ECL) followed by autoradiography.

### Immunohistochemistry

Cells were seeded on cover slips. To induce cilia formation some cells were serum-starved for 48 h. Cells were then washed with PBS and fixed with 4% PFA for 15 min at room temperature. After washing fixed cells three times with PBS, cells were blocked in blocking solution (10% goat serum, 5% milk, 0.1% Triton X-100 in PBS) for 1 hour at room temperature. Next, cells were incubated with primary antibodies against the IR_α_ (1:250, Santa Cruz) or IR_β_ (1:250, Santa Cruz) with or without the anti-Ac-α Tubulin antibody (1:250, Santa Cruz) for 2 h at room temperature followed by 3 washes in PBS, for 5 min each. Cells were then incubated with secondary immunofluorescent antibodies, goat-anti-rabbit Alexa488 and goat-anti-mouse Alexa568 (Invitrogen) for 1 h at room temperature followed by washing as above. Finally cover slips were mounted using VectaShield mounting medium with DAPI. Images were visualized using confocal microscopy (Zeiss 710) and analyzed using ImageJ software.

### Glucose and insulin measurements

Fasting blood glucose measurements were taken using a glucometer (OneTouch Ultra) with a blood sample taken from a tail snip. Insulin levels were measured using an ELISA kit (Crystal Chem). The mice were fasted overnight followed by cardiac puncture for blood collection. Plasma was isolated using 0.5 M EDTA and centrifugation to separate plasma from red blood cells. Insulin concentration in the plasma was measured by radioimmunuassay using a commercially available kit (Crystal Chem Inc.).

### Glucose and insulin tolerance tests

Glucose tolerance test (GTT) and insulin tolerance test (ITT) were performed as previously described [45]. Four to five month old mice were fasted overnight (GTT) or for 5 h (ITT) in a clean cage. Body weight and basal blood glucose measurements were taken before intraperitoneal injection of glucose (2 g/kg) or insulin (1 U/kg). Blood glucose measurements were assessed at multiple time points during the two-hour test.

### Statistical analysis

All data are expressed as means ± SEM. A two-way ANOVA was used to compare BBS mice to wild type (WT) littermates for GTT and ITT analyses and insulin signaling assay. The first factor was genotype, and the second factor was time point for GTT and ITT analyses, while the second factor was treatment for insulin signaling. Multiple-comparison testing following two-way ANOVA was performed using a Bonferroni t-test. One-way ANOVA was used to compare more than two groups, such as baseline blood glucose and plasma insulin. Rank-ANOVA was used whenever the data did not follow a normal distribution. A two-tailed p-value < 0.05 was considered significant for all analyses.

## Supporting Information

S1 Fig Interaction between BBS17 and IR and efficacy of shRNA-mediated BBS gene silencing. A) Co-immunoprecipitation of the Flag-tagged BBS17 with the β subunit of the IR in protein lysates from HEK293T cells. **B**) Ability of the endogenous IR (β subunit) to pull down the endogenous BBS17 protein in mouse brain lysates. **C-D**) Efficiency of the shRNA targeting the *Bbs1* (**C**) or *Bbs2* (**D**) genes in HEK293T cells to knockdown the expression of the proteins. HA-tagged system was used for immunoblot recognition of BBS1 and BBS2 proteins.
(TIF)

S2 Fig BBSome proteins are not required for surface expression of transferrin receptor (TfR). **A**) Silencing *Bbs1* or *Bbs2* genes does not affect the amount of transferrin receptor at the cell surface in HEK293T cells. **B**) MEF of *Bbs1* ^*M390R/M390R*^ knock-in (KI) mice have unchanged TfR levels relative to wild type (WT) littermates. Bar graph data are expressed as means ± SEM.
(TIF)

S3 Fig BBS chaperonin proteins are required for membrane localization of the IR. Surface expression of the insulin receptor was reduced in HEK293T cells in which *Bbs6* (**A**) or *Bbs12* (**B**) genes were silenced using GFP-tagged shRNA. Note that cell surface expression of the insulin receptor was selectively reduced in the transfected cells (expressing GFP). The nuclei were stained with DAPI.
(TIF)

S4 Fig Insulin receptor does not localize to the cilia in 3T3L1 fibroblasts when examined by immunohistochemistry. The signal for the cilium marker (acetylated-α Tubulin) is distinct from the signal for IR. Cells were serum-starved for 48 hours to stop proliferation and induce cilium formation. Antibody against acetylated-α Tubulin with Alexa Fluor^568^ secondary antibody was used to label cilium and antibody against IR β-subunit with Alexa Fluor^488^ secondary antibody was used to label the IR. The nuclei were stained with DAPI.
(TIF)

S5 Fig Calorie-restricted (CR) *Bbs2* ^*-/-*^, *Bbs4* ^*-/-*^, *Bbs6* ^*-/-*^ mice have normal body weights relative to wild type (WT) mice. Individuated housed *Bbs2* ^−/−^, *Bbs4* ^−/−^ and *Bbs6* ^−/−^ mice were given 75–80% of the chow pellets normally consumed daily by sex- and age-matched WT mice. This calorie restriction protocol effectively prevented obesity in BBS mice. Data are expressed as means ± SEM.
(TIF)
