# Supplementary figures and images for: Regulation of Insulin Receptor Trafficking by Bardet Biedl Syndrome Proteins
Source: PLoS Genet. 2015 Jun 23;11(6):e1005311. doi: 10.1371/journal.pgen.1005311 (PMC4478011; doi:10.1371/journal.pgen.1005311)

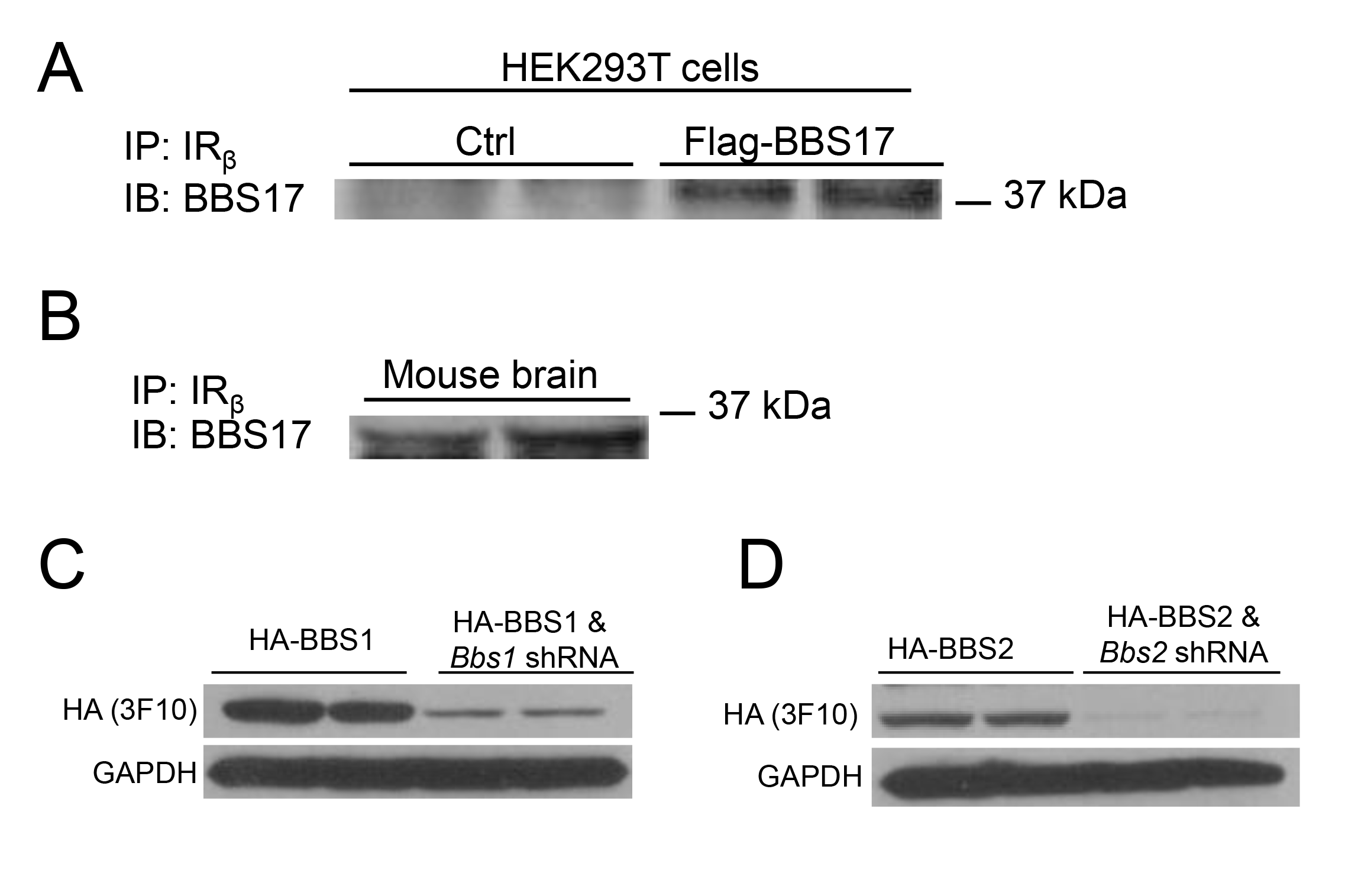

Supplement: S1 Fig — A) Co-immunoprecipitation of the Flag-tagged BBS17 with the β subunit of the IR in protein lysates from HEK293T cells. B) Ability of the endogenous IR (β subunit) to pull down the endogenous BBS17 protein in mouse brain lysates. C-D) Efficiency of the shRNA targeting the Bbs1 (C) or Bbs2 (D) genes in HEK293T cells to knockdown the expression of the proteins. HA-tagged system was used for immunoblot recognition of BBS1 and BBS2 proteins. (TIF) [file pgen.1005311.s001.tif]

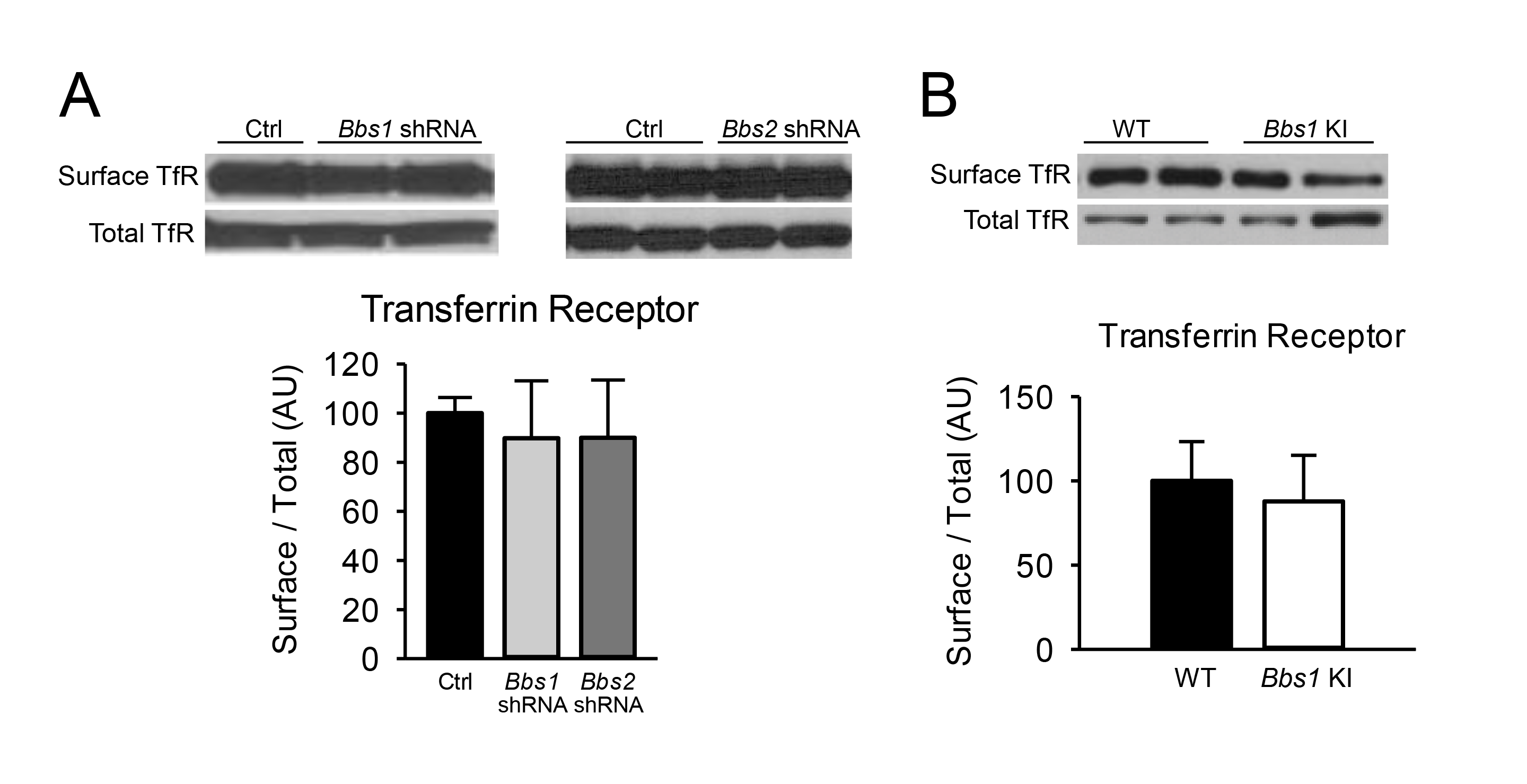

Supplement: S2 Fig — A) Silencing Bbs1 or Bbs2 genes does not affect the amount of transferrin receptor at the cell surface in HEK293T cells. B) MEF of Bbs1 M390R/M390R knock-in (KI) mice have unchanged TfR levels relative to wild type (WT) littermates. Bar graph data are expressed as means ± SEM. (TIF) [file pgen.1005311.s002.tif]

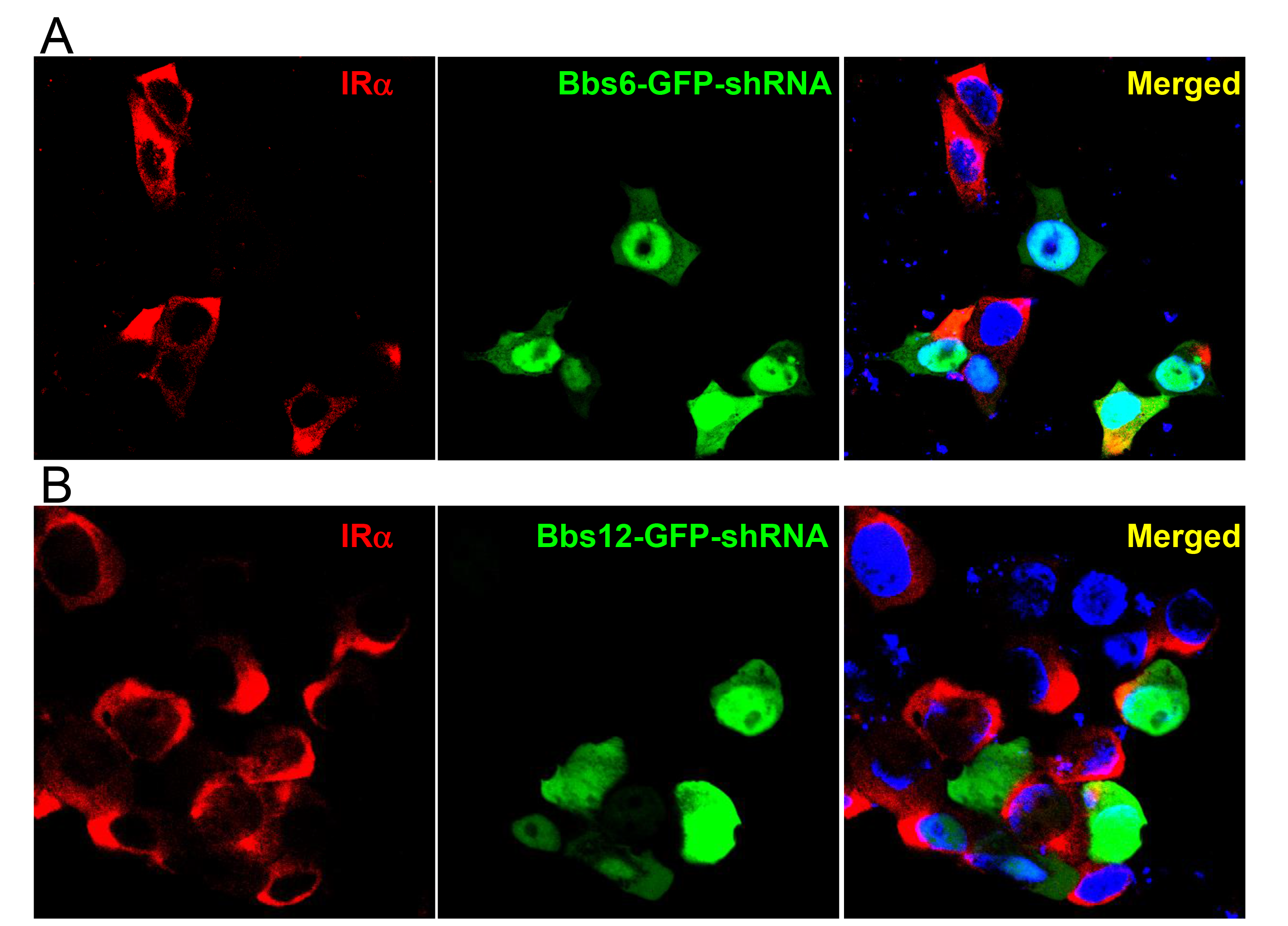

Supplement: S3 Fig — Surface expression of the insulin receptor was reduced in HEK293T cells in which Bbs6 (A) or Bbs12 (B) genes were silenced using GFP-tagged shRNA. Note that cell surface expression of the insulin receptor was selectively reduced in the transfected cells (expressing GFP). The nuclei were stained with DAPI. (TIF) [file pgen.1005311.s003.tif]

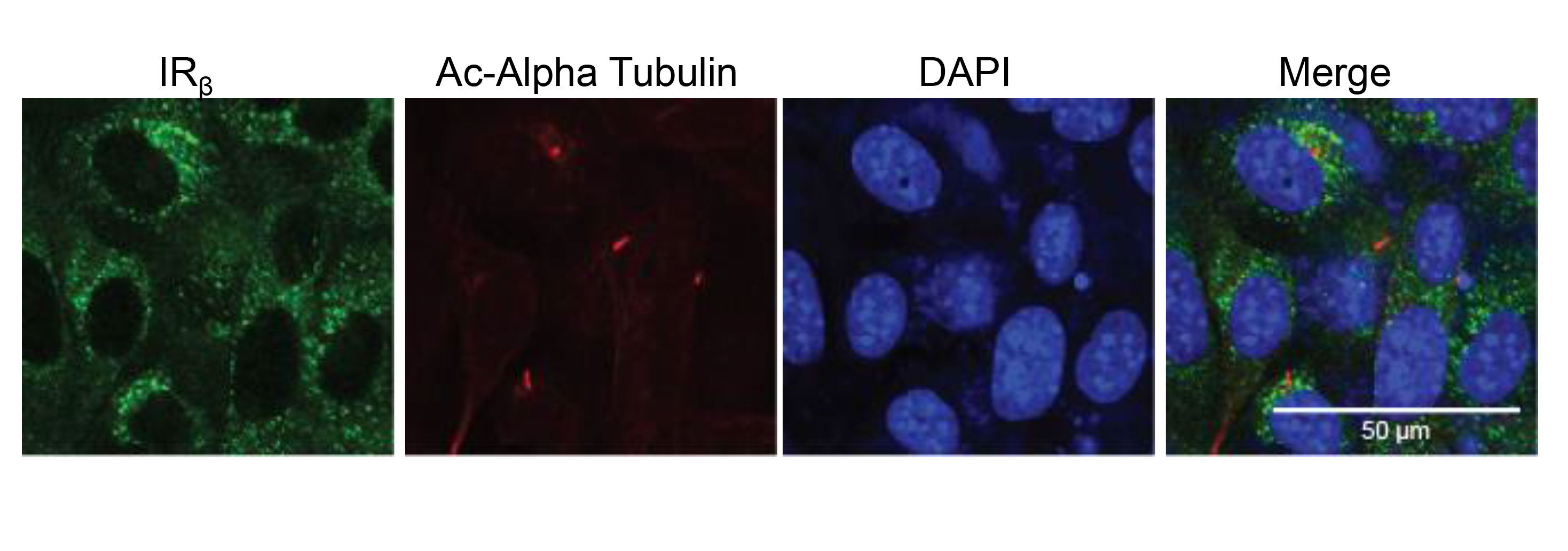

Supplement: S4 Fig — The signal for the cilium marker (acetylated-α Tubulin) is distinct from the signal for IR. Cells were serum-starved for 48 hours to stop proliferation and induce cilium formation. Antibody against acetylated-α Tubulin with Alexa Fluor568 secondary antibody was used to label cilium and antibody against IR β-subunit with Alexa Fluor488 secondary antibody was used to label the IR. The nuclei were stained with DAPI. (TIF) [file pgen.1005311.s004.tif]

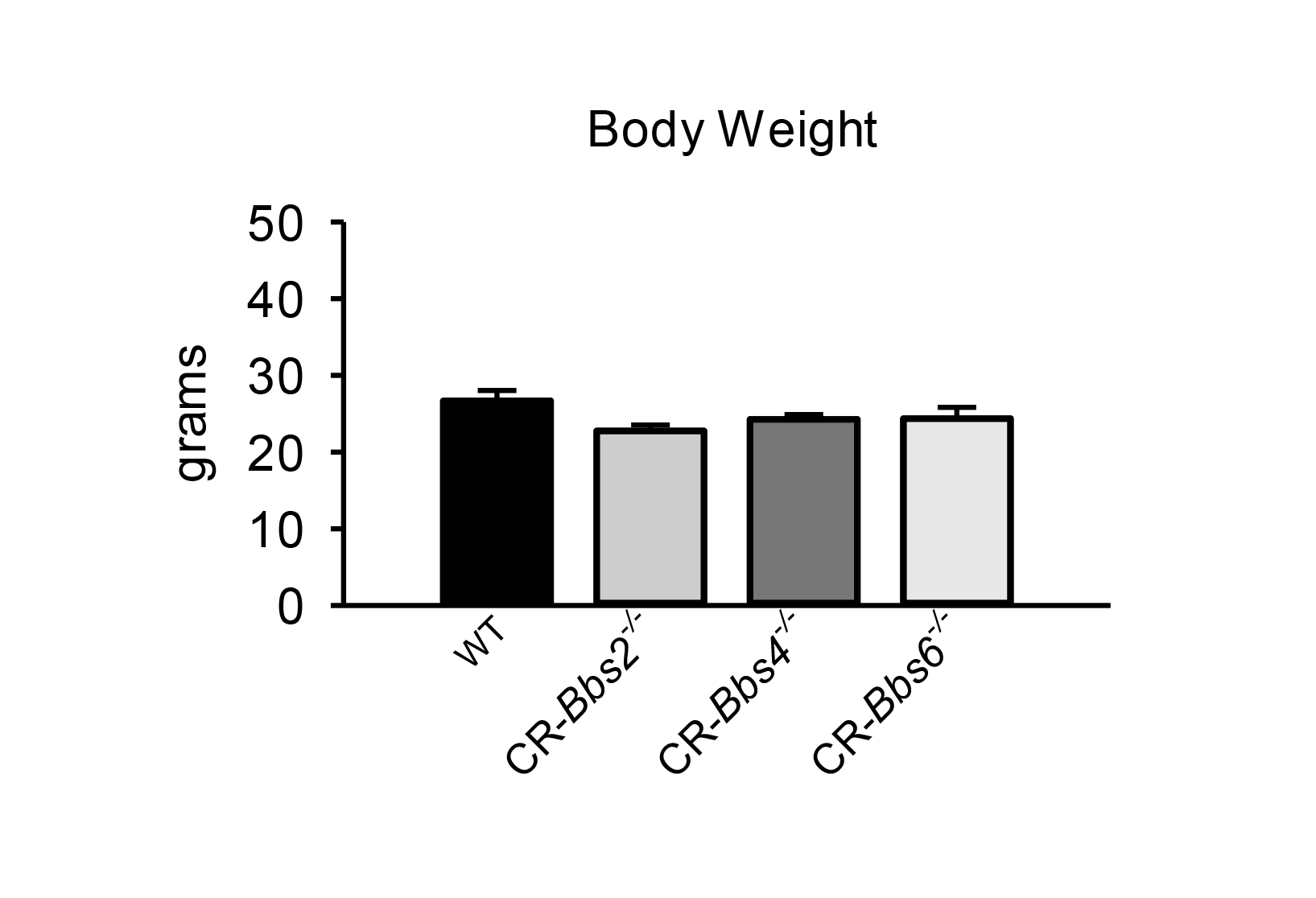

Supplement: S5 Fig — Individuated housed Bbs2 −/−, Bbs4 −/− and Bbs6 −/− mice were given 75–80% of the chow pellets normally consumed daily by sex- and age-matched WT mice. This calorie restriction protocol effectively prevented obesity in BBS mice. Data are expressed as means ± SEM. (TIF) [file pgen.1005311.s005.tif]
